# Younger Age Is Associated with Favorable Outcomes in Adult Dogs with Hemangiosarcoma Receiving Adjuvant Doxorubicin Chemotherapy: Results from the PRO-DOX Study

**DOI:** 10.21203/rs.3.rs-6573099/v1

**Published:** 2025-05-05

**Authors:** Antonella Borgatti, Brian D. Husbands, Aaron L. Sarver, Jeremy M. Chacón, Todd E. DeFor, Aaron Rendahl, Michael S. Henson, Jaime F. Modiano, Kathleen M. Stuebner, Amber L. Winter, Heather Scavello, Sara Pracht, Andrea Chehadeh, Kelly Bergsrud, Caitlin Feiock, Bailey Anderson, Sarah Kenney, Matthew J. Atherton, Pascale C. Salah, Jennifer Mahoney, David R. Brown, Michael O. Childress, Erin B. Dickerson

**Affiliations:** University of Minnesota; The Ohio State University; University of Minnesota; University of Minnesota; University of Minnesota; University of Minnesota; University of Minnesota; University of Minnesota; University of Minnesota; University of Minnesota; University of Pennsylvania; University of Minnesota; University of Minnesota; University of Minnesota; University of Minnesota; University of Minnesota; University of Minnesota; University of Pennsylvania; University of Pennsylvania; Tufts University; University of Minnesota; Purdue University; University of Minnesota

**Keywords:** Aging, canine, DNA damage response, hemangiosarcoma, doxorubicin, immunity, propranolol, senescence

## Abstract

**Background:**

Canine hemangiosarcoma is a common and aggressive vascular malignancy predominantly affecting dogs over six years of age. Despite surgical resection followed by adjuvant chemotherapy, median survival remains around 4–6 months. Propranolol, a beta-adrenergic receptor (b-AR) antagonist, has shown efficacy in human angiosarcoma, a tumor with similar clinical and morphological characteristics, when combined with chemotherapy.

**Methods:**

To determine if propranolol could be repurposed as an effective adjunct to chemotherapy, we conducted a phase I clinical study evaluating the safety and efficacy of propranolol combined with doxorubicin (PRO-DOX) in 20 dogs with stage 1 or stage 2 splenic hemangiosarcoma.

Plasma from 19 dogs was analyzed for propranolol pharmacokinetics and RNA was extracted from tumors from 13 of the dogs for transcriptional profiling.

**Results:**

Although propranolol did not appear to influence treatment outcomes, our results revealed long-term survival in young adult dogs (less than 6 years of age), suggesting the possibility of a better response to doxorubicin. Faster clearance of 4-OH propranolol also correlated with long-term survival in younger dogs, but this appeared to be associated with drug metabolism due to age rather than effects of the drug on survival outcomes. Gene expression analysis identified distinct age-associated tumor signatures, with young dogs exhibiting increased immune-related gene expression and older dogs showing elevated expression of genes associated with the cell cycle and the DNA damage response and repair.

**Conclusions:**

These findings highlight several hallmarks of cellular aging in hemangiosarcoma that may influence treatment responses and long-term survival. Our findings suggest that young adult dogs with splenic hemangiosarcoma treated with doxorubicin have a better prognosis and underscore the need for further research into age-related molecular mechanisms of disease. These insights could refine therapeutic strategies and clinical decision-making in hemangiosarcoma management.

## Background

Canine hemangiosarcoma is a common and highly aggressive vascular malignancy in dogs, with an estimated incidence exceeding 50,000 cases annually (1). Tumors consist of irregularly formed and leaky vessels that can impede normal blood flow, leading to necrosis and extensive hemorrhage (1). The most affected sites include the spleen, right auricle of the heart, liver, and skin or subcutaneous tissue (2, 3). Although the addition of doxorubicin-based chemotherapy alone or following surgical resection has modestly extended median overall survival (4), clinical outcomes have remained poor, with the average life expectancy following standard treatment at six months or less with approximately 10% of dogs surviving beyond one year (4–6).

The limited success of broadly applied chemotherapy for treating hemangiosarcoma is likely influenced by several factors, including the heterogeneity of the disease where responses may be driven by distinct gene expression and mutation profiles (7–12). Age-related factors that influence disease progression and potentially treatment responses may also be key determinants of outcomes, as hemangiosarcoma predominantly affects older dogs, with a median onset at 10 years (13). Treatment decisions and care in older dogs can be complicated by comorbidities, both diagnosed and undiagnosed, that may affect tolerance and responses to doxorubicin-based chemotherapy. Treating younger dogs raises concerns about potential long-term complications, such as cardiotoxicity, which can manifest months or years after treatment (14, 15). The increased incidence of cancer with age also correlates with a decline in immune function, dampening local and systemic anti-tumor immunity (16).

A rare, but similar disease, called angiosarcoma, affects humans. Angiosarcomas are aggressive soft tissue sarcomas predominantly found in adults over the age of 65 (17, 18). About 60% of cases manifest as cutaneous lesions on the head and neck, while the remainder are largely found in the liver, spleen, heart, and bones (19). Angiosarcoma shares its clinical presentation and morphology with canine hemangiosarcoma (1); however, this disease is diagnosed in fewer than 1000 patients each year in the United States (18). Despite multimodal treatment strategies, including surgery, radiotherapy, and chemotherapy, the five-year survival rate for angiosarcoma patients is approximately 30% and is further reduced to 10–12 months in cases of metastatic disease (20, 21). The high incidence of canine hemangiosarcoma, combined with its similarities to angiosarcoma, suggests it might have utility as a spontaneous cancer model, specifically to address age-related vulnerabilities and response to treatment (1, 22–24).

Recent studies have shown that the non-selective beta-adrenergic receptor (b-AR) antagonist propranolol exhibits promising clinical efficacy in angiosarcoma when combined with conventional chemotherapy. This combination has led to remarkable responses in some patients, including those with metastatic disease (25–29). b-ARs are highly expressed in angiosarcoma (30), and we have reported their expression in canine hemangiosarcoma (31). Previous studies have also shown propranolol inhibits proliferation and induces apoptosis in angiosarcoma and canine hemangiosarcoma cell lines and inhibits the tumor growth in a preclinical angiosarcoma model (30, 31). Additionally, we reported synergy between propranolol and doxorubicin in angiosarcoma and hemangiosarcoma cells, supporting its potential to enhance chemotherapy efficacy. β-AR signaling also plays a critical role in immune regulation, with β-AR antagonism reducing myeloid-derived suppressor cells (MDSCs) in tumors and enhancing CD8 + T cell activation and infiltration, thereby supporting anti-tumor responses (32). Additionally, exhausted CD8 + T cells exhibit increased expression of β1-AR, driving their progression toward dysfunction (33). Given that immune decline is a hallmark of aging and contributes to cancer risk, these findings provided a strong rationale for repurposing propranolol as an adjunct to chemotherapy for treating hemangiosarcoma in dogs due to its dual anti-tumor and immune-enhancing effects.

**Figure 1:**
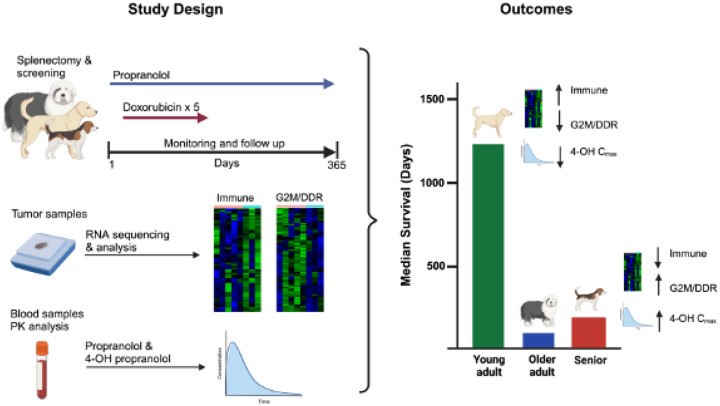
Summary of the PRO-DOX study design and outcomes. The schema illustrates the clinical trial design and outcomes for 20 dogs enrolled in PRO-DOX and the correlative RNA-sequencing and pharmacokinetic studies performed on tumors and blood samples, respectively. Figure created with BioRender.

Here, we investigated the safety and efficacy of combining propranolol with doxorubicin chemotherapy in pet dogs diagnosed with splenic hemangiosarcoma (Fig. 1). Our results suggest the addition of propranolol may not improve overall survival. Instead, they suggest an age-related difference in prognosis, with young adult dogs experiencing a significant long-term survival advantage when treated with surgery and doxorubicin. They also reveal potentially important and targetable age-related differences in gene expression in canine hemangiosarcoma.

## Methods

### Clinical study

A phase I study was designed using a continuous reassessment model for the clinical evaluation of propranolol in combination with doxorubicin chemotherapy (PRO-DOX) (34). Dogs enrolled in PRO-DOX were client-owned dogs presenting at University of Minnesota (MN) Veterinary Medical Center (VMC), University of Pennsylvania (UP) Ryan Veterinary Hospital, and Purdue University (PD) Small Animal Hospital. All owners provided written informed consent prior to enrollment. The study was managed by the Clinical Investigation Center at the University of Minnesota in compliance with principles of Good Clinical Practice and conducted with the approval of the Institutional Animal Care and Use Committee (IACUC) for the University of Minnesota, the University of Pennsylvania, and Purdue University. Study data were collected and managed using Research Electronic Data Capture (REDCap) (35).

Inclusion criteria included a confirmed diagnosis of stage 1 (no evidence of tumor rupture) or stage 2 (evidence of tumor rupture) splenic hemangiosarcoma by histopathology with no evidence of gross regional or distant metastatic disease. All dogs received a physical examination and laboratory testing that included a complete blood count, serum biochemical profile, coagulation profile and urinalysis. Thoracic radiography, abdominal ultrasonography, and an echocardiogram were performed prior to enrollment. Dogs were required to have constitutional clinical signs of 0 or 1 per Veterinary Cooperative Oncology Group (VCOG) criteria (36), life expectancy greater than 12 weeks with treatment, and adequate hematologic, renal, and hepatic function for enrollment. Eligibility was restricted to dogs that had undergone splenectomy within 30 days before starting treatment and those weighing at least 20 kg. Dogs receiving any herbal or complementary treatments were excluded unless treatments were given for less than 24 hours followed by a 72 hour wash out period before starting PRO-DOX.

Dogs began treatment with propranolol after splenectomy but before doxorubicin chemotherapy. Three target dose cohorts were used for the study based on the likely effective dose range (0.7–1.5 mg/kg) identified for human patients with angiosarcoma treated with propranolol (26, 29, 37). The trial was conducted using intra-subject escalation to the highest dose using three cohorts: cohort 1 (0.8 mg/kg), cohort 2 (1.0 mg/kg) and cohort 3 (1.3 mg/kg) with predefined criteria for toxicity (no dose-limiting adverse events within the first 21 days). Dose limiting toxicity (DLT) was defined as any hematologic adverse event (AE) > grade 3, non-hematologic AE > grade 2 or cardiovascular AE prior to obtaining the day 3 post-target dose. Propranolol was administered orally three times per day starting at a dose of 0.5 mg/kg. Based on a Bayesian framework, dose-levels were allowed to increase or decrease based on initial clinical estimates of DLT rates in addition to sequential data driven estimates. Each new cohort of two dogs was sequentially assigned to the most appropriate dose (either one step down, the same dose, or one step up) based on the assessment of adverse events. Adverse events were graded according to VCOG-CTCAE criteria (36). Reductions in dosage and/or frequency were permitted to manage adverse events attributed to propranolol. Adverse events related to doxorubicin were treated with supported care.

All dogs began doxorubicin chemotherapy on day 12 after enrollment. Doxorubicin was administered at 30 mg/m^2^ intravenously every three weeks for five cycles. Propranolol was continued during chemotherapy and sustained for up to one year at the cohort dose. Owners were given the option to continue propranolol beyond the 1-year trial period. Assessment of disease progression included a physical exam, complete blood count, serum biochemical profile, urinalysis and repeat thoracic radiographs and abdominal ultrasound prior to doxorubicin treatments three through five. An identical assessment was completed at months six, nine, and 12. Survival time was measured from the date of splenectomy to the time of death and was censored at the time of last contact for dogs surviving at the time of analysis. Two historical control groups were combined for comparison. The first consisted of 21 dogs with stage1 or stage 2 splenic hemangiosarcoma treated with surgery followed by doxorubicin-based chemotherapy at the University of Minnesota between 2005 and 2011 (38, 39). The second consisted of 19 dogs with stage 1 or stage 2 hemangiosarcoma treated with surgery followed by doxorubicin chemotherapy at the University of Pennsylvania Ryan Veterinary Hospital between 2013 and 2019 (40).

### RNA sequencing and bioinformatics analysis

RNA was extracted from five 10 μm sections obtained from formalin-fixed paraffin-embedded (FFPE) samples from 13 out of 20 dogs enrolled in PRO-DOX using a PureLink FFPE Total RNA Isolation Kit (Cat# K1560–02, ThermoFisher Scientific, Waltham, MA) according to the manufacturer’s instructions. Samples were treated with DNase I to generate DNA-free total RNA. Extractions were performed by the University of Minnesota Genomics Center (UMGC) and the total RNA from each sample was quantified and assessed for quality. RNA integrity numbers (RIN) ranged from 2 to 7.1. Because lower quality RNA was expected from FFPE samples, a RIN value of 2 was accepted as the lowest limit for RNA sequencing (RNA-seq)(2 × 50 base paired-end on a NovaSeq S2). RNA-seq was performed by the UMGC.

Gene cluster expression summary score (GCESS) analyses were performed as previously described on average linkage hierarchical clustered data (41). To process the data for GCESS, a quality control analysis of RNA-seq FASTQ data was performed using FastQC software (v0.11.5). FASTQ data were trimmed with Trimmomatic (v0.33.0), and Kallisto (v0.43.0) was used for pseudoalignment and quantifying transcript abundance. Sequencing reads were aligned to the canine reference genome (CanFam6). Transcript abundance counts were generated, and quantile normalized to correct for differences in sequence counts. Clusters of genes were identified based on patterns of correlated expression (e.g. those associated with G2/M checkpoints). Following mean-centering and log_2_-transformation, individual gene values in each cluster were added together, resulting in a single summary score for the cluster that is reflective of the overall degree of gene expression.

To process the RNA-seq data for differential expression and gene set enrichment, the raw FASTQ files were processed using the CHURP (v. 1.0) pipeline (42) using FastQC (v0.11.5), Trimmomatic, HISAT2, and featureCounts to generate a counts matrix. The CanFam6 genome assembly was used for mapping and feature counting. Raw gene counts were imported into R. Because RNA-seq libraries were sequenced in two different batches, the batch effect was removed by applying ComBat_seq (in sva 3.52.0) (43). Next, edgeR (v. 4.2.1) was used to filter out genes shorter than 500bp or that had fewer than 30 counts in 70% of the samples. The samples were divided into three groups based upon their overall survival. edgeR::estimateDisp was used to determine gene variances, and then edgeR::glmQLFit was used to fit the model. The contrast between the long-lived and short-lived dogs was calculated using edgeR::glmQLFTest. For gene set enrichment analysis (GSEA), the genes were ranked by −log10(PValue) * sign(log2FC) from the long vs. short contrast. This gene list was input into gseGO in clusterProfiler (v 4.12.6) with an adjusted p-value cutoff of 0.05. The pheatmap package (v. 1.0.12) was used to generate heatmaps (44, 45).

### Pharmacokinetics

Plasma was collected from 19 of 20 dogs for pharmacokinetics. One dog did not survive long enough for sample collection. Samples for propranolol analysis were collected on the morning of day 11 of the protocol. To obtain a baseline level, propranolol was withheld by the owners in the morning and plasma was collected by clinical staff. Dogs were then given propranolol based on their enrolled cohort, and plasma was obtained at 30, 60, 90, 120, 240, and 360 minutes. Samples were also collected at 24 hours on day 12. All samples were frozen and stored at −80°C until analysis by the Clinical Pharmacology Analytical Services (CPAS) core, University of Minnesota, College of Pharmacy. The concentrations of propranolol and 4-hydroxypropranolol (4-OH propranolol), a main metabolite produced after oral administration of propranolol that maintains antagonistic effects on β-ARs (46), were determined using specific LC/MS methods previously developed by the CPAS core.

### Cell culture and reagents

The DHSA-m1426 cell line was derived from 1426-DHSA cells (available from Kerafast, Boston, MA) injected into immunocompromised mice. Cells were maintained in Ham’s F12 medium supplemented with 10% fetal bovine serum (Atlas Biologicals), 1% HEPES (Cat# 15630080, ThermoFisher Scientific), 0.05 mg/mL Endothelial Cell Growth Supplement (Cat# 356006, Corning, Corning NY, USA), 100 μg/mL Primocin (InvivoGen, San Diego, CA), and 0.01 mg/mL heparin (Cat# H3149, Millipore Sigma, Burlington, MA) at 37°C in a humidified 5% CO_2_ atmosphere. Mycoplasma contamination was tested regularly and the cell line authenticated using CellCheck^™^ Canine STR profiling test (IDEXX BioAnalytics, Columbia, MO).

### Drug synergy assay

DHSA-m1426 cells were plated at 5,000 cells per well in duplicate 96-well plates in 100 μL of cell culture medium and allowed to adhere overnight. Cells were treated with combinations of doxorubicin (7.8–500 nM) (Cat# S1208, Selleckchem, Houston, TX, USA) and propranolol (25–200 μM) (Cat# 0624, Bio-Techne Corporation, Minneapolis, MN, USA) for 72 hours and viability was determined using a colorimetric MTS reagent (Cell Titer 96^®^ Aqueous Non-Radioactive Cell Proliferation Assay, Promega, Madison, WI), according to the manufacturer’s instructions. Combination index (CI) values for all tested drug concentrations were determined according to the method of Chou and Talalay (47) and calculated using Compusyn software (Combosyn Inc., NJ, USA), as described (31). The CI theorem quantitatively defines the CI values as additivity (0.9 ≤ CI ≤ 1.1), synergy (CI < 0.9), and antagonism (CI > 1.1).

### Tumor xenograft

Female immunodeficient mice (bg/nu/xid, NIH-III) age 6–7 weeks (Charles River Laboratories, Wilmington, MA) were injected subcutaneously in the right flank with 5×10^6^ DHSA-m1426 cells in a total volume of 200 μL mixed in a 1:1 ratio with Matrigel (Cat# 354234, Corning, Corning, NY). Once tumor volumes reached approximately 200 mm^3^, mice were randomly divided into four groups (10 mice/group) and treated with doxorubicin (4 mg/kg) once per week by intraperitoneal injection, propranolol (7 mg/kg) *ad libitum* via the drinking water, or with doxorubicin and propranolol. Untreated mice were used as controls. The animals were monitored daily. The weight of the animals and tumor size were measured every two to three days. To determine tumor volume, two perpendicular diameters were measured with calipers, and tumor volume was calculated using the formula (D × d^2^)π/6, with D as the largest and d as the smallest diameters (48). All mice were euthanized once a tumor in any group reached a volume of 2 cm^3^. Animal housing, handling, and euthanasia procedures were conducted in compliance with the guidelines and approval of the University of Minnesota IACUC.

### Statistical analysis

Baseline characteristics were summarized using descriptive statistics and compared between PRO-DOX treated dogs and the historical comparison group (control group) using a Chi-squared test for categorical variable comparisons, and an unpaired *t* test for continuous variable comparisons. For the comparison of mean values between three or more groups, a one-way ANOVA with Tukey post-hoc test was performed. The primary survival outcome was time until hemangiosarcoma-related mortality. Survival time for all dogs was measured from the date of surgery until the time of death. Dogs that died for reasons other than hemangiosarcoma were censored at the time of death. Kaplan Meier plots were used to summarize overall survival, and statistical comparison between groups (PRO-DOX versus control) was performed by log rank test. A Fisher’s exact test was used to evaluate exceptional survival. Correlation analyses between survival and drug concentration values were performed using Spearman’s correlation with survival censored at one year. Statistical analyses were performed using Graph Pad Prism software (v10.4.1). and R version 4.4.1 (https://www.R-project.org).

## Results

### Younger age is associated with durable remissions in dogs receiving adjuvant doxorubicin chemotherapy for splenic hemangiosarcoma.

The safety and efficacy of propranolol in combination with doxorubicin were assessed in dogs diagnosed with splenic hemangiosarcoma using an adaptive phase I trial design. Twenty dogs diagnosed with splenic hemangiosarcoma meeting eligibility criteria were enrolled. Initially, two dogs were enrolled in the 0.8 mg/kg dose cohort, followed by two dogs each in the 1.0 and 1.3 mg/kg dose cohorts. As no severe adverse events were observed during dose escalation, the remaining 14 dogs were enrolled in the highest dose cohort of 1.3 mg/kg. The treatment timeline is illustrated in Figure S1A. Baseline characteristics for all dogs treated with propranolol plus doxorubicin and those in the comparison (historical control) group receiving doxorubicin-based standard of care are presented in Table 1.

Propranolol monotherapy (days 1–11) was well tolerated across all cohorts. Only two mild adverse events, diarrhea and a persistent cough, were reported during the dose escalation phase. Importantly, none of the dogs experienced significant hypotension (Fig S2). Neutropenia attributable to doxorubicin was limited to two dogs in cohort 3. Median survival for the 20 dogs treated with propranolol and doxorubicin was 134 days compared with 152 days for the comparison group (n=40) of dogs treated with doxorubicin-based standard of care (Fig S1B). There was no significant difference in overall survival between the two groups (p=0.59; HR 0.9; 95% CI, 0.51–1.48).

The lack of an additional benefit in dogs was unexpected, given previously reported studies suggesting propranolol augments chemotherapy in angiosarcoma patients (26–29). To further investigate these findings, we inoculated immunocompromised bg/nu/xid mice with a canine hemangiosarcoma cell line, DHSA-m1426, to generate subcutaneous xenografts for monitoring drug responses. Since our prior work demonstrated propranolol enhances the activity of doxorubicin in hemangiosarcoma cell lines (31) we first validated this interaction in DHSA-m1426 cells using an established *in vitro* assay to quantify the synergistic interaction between propranolol and doxorubicin (Fig S3A). We then treated tumor-bearing mice with propranolol (7 mg/kg) administered via the drinking water and doxorubicin (4 mg/kg) given by weekly intraperitoneal injections, either as a monotherapy or in combination with propranolol. Both doxorubicin and propranolol independently reduced tumor growth in mice (Fig S3B, C); however, the combination treatment did not produce greater tumor inhibition than either drug alone, aligning with our clinical findings.

The use of immunocompromised bg/nu/xid mice in the DHSA-m1426 model prevented us from evaluating the contribution of T cells to the anti-tumor response, a significant limitation since previous studies have shown the anti-tumor effects of propranolol rely on T cells (49, 50). The absence of this immune component in our preclinical model may explain, at least in part, the observed lack of additional benefit with propranolol in dogs as propranolol may fail to activate a robust immune response in dogs similar to those observed in immunocompetent mice. This limitation may also be exacerbated in older dogs, where age-related immune decline could further impair the potential for propranolol to enhance immune-mediated anti-tumor responses.

**Figure 2:**
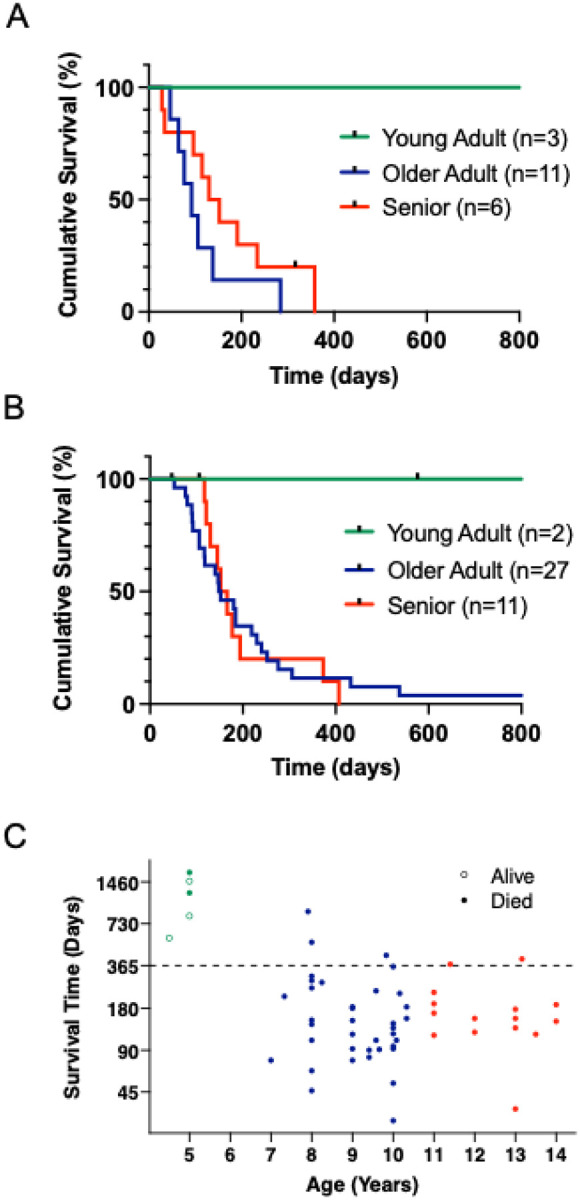
Young adult dogs treated with doxorubicin-based therapies exhibit prolonged survival versus older adult and senior dogs. **A)** Kaplan–Meier curves for dogs enrolled in the PRO-DOX study (n=20) or **B)** historical controls (n=40) grouped by age, Young Adult (YA) < 7 years; Older Adult (OA) ^3^ age 7 to < 11 years; Senior (S) 3 age 11 years). **C)** Distribution of all dogs enrolled in the PRO-DOX and control groups by age and survival time in days is shown. A Fisher’s exact test (p<0.001) was used to compare the survival rates between YA (green) (5/5) and OA (blue) and S (red) dogs (5/52) focusing on exception survival (>365 days). Dogs that died of causes other than hemangiosarcoma before day 365 (n=3) were excluded from the analysis.

Although the overall survival outcomes between the PRO-DOX and the control groups did not differ significantly, we observed that all three dogs diagnosed at age five in the PRO-DOX group survived for more than two years. These outcomes were unrelated to the propranolol dose, as each of these dogs was enrolled in a different cohort: MN-01 (cohort 1, 0.8 mg/kg), PD-01 (cohort 2, 1.0 mg/kg), and MN-05 (cohort 3, 1.3 mg/kg). Furthermore, survival times for the older adult and senior dogs were not age-dependent, as all but one dog ultimately succumbed to progressive disease. Two young adult dogs in the control group also exhibited extended survival times of 576 days (lost to follow up) and 1704 days. Because these data suggest that a younger age at diagnosis may predict a favorable outcome, we divided the dogs into three age groups—young adult, older adult, and senior dogs—and compared their median survival times (Fig 2A, B). Age groups were informed by veterinary life stage guidelines, canine mortality data, and studies on epigenetic aging (51–54): senior dogs were defined as 11 years or older, older adults as age seven to less than 11 years, and young adults as younger than seven years. The survival time for the young adult dogs in the PRO-DOX study was significantly longer (p = 0.009) compared to the older adult and senior groups; however, there was not a pronounced difference in overall survival time between the older adult and the senior dogs (p=0.2). For the control group, the survival time also differed significantly between the young adult dogs and the older adult and senior groups (p=0.03); a difference was not observed between the older adult and senior dogs (p=0.93). We then looked to see if there was a significant association between age and exceptional survival (defined as survival greater than 365 days) by comparing the YA dogs from the OS and S dog from both the PRO-DOX and control groups (Fig. 2C). Young age at diagnosis was associated with survival (p < 0.001), indicating that age at diagnosis might influence prognosis, perhaps via responses to doxorubicin-based treatments, with young adult dogs appearing to benefit disproportionately.

### Propranolol pharmacokinetics are impacted by age.

Although the observed survival advantage in the young adult dogs could largely be attributed to doxorubicin rather than propranolol, we conducted further studies to assess whether propranolol conferred any additional survival benefit. Since propranolol’s pharmacokinetics in human subjects are age-dependent, where younger individuals exhibit increased metabolic clearance (55, 56), we sought to investigate whether similar age-related differences exist in dogs and whether these differences correlate with overall survival. Additionally, we examined the pharmacokinetics for 4-OH propranolol, the primary metabolite of propranolol in dogs, which has a similar high binding affinity and antagonist activity for b-ARs (46, 57).

**Figure 3:**
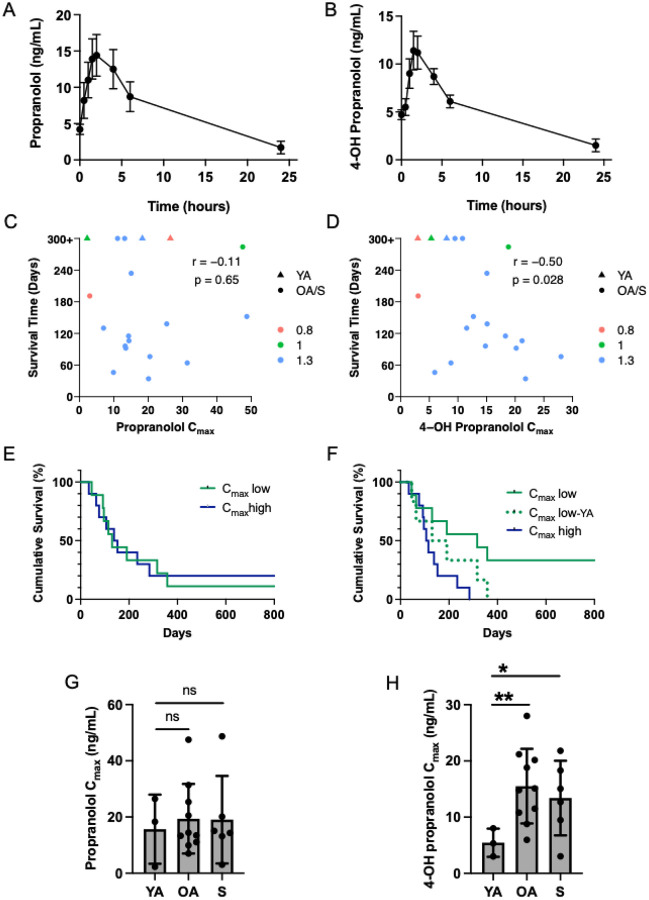
4-OH propranolol pharmacokinetics are associated with age. Mean (± S.E.M) plasma concentrations of **A)** propranolol and **B)** 4-OH propranolol over time following oral administration of propranolol three times daily for 11 consecutive days (n=19). **C)** Correlation between the propranolol C_max_ and survival (Spearman *r* = −0.11) and **D)** 4-OH propranolol C_max_ and survival (Spearman *r* = −0.50). E) Kaplan–Meier survival analysis for dogs (n=19) according to C_max_ values for propranolol. C_max_low reflects 50% of the dogs with lowest C_max_ values for propranolol; C_max_high reflects 50% of the dogs with the highest C_max_ values, p=0.96. **F)** Kaplan–Meier survival analysis for dogs (n=19) according to C_max_ values for 4-OH propranolol. C_max_low reflects 50% of the dogs with lowest C_max_ values for 4-OH propranolol; C_max_high reflects 50% of the dogs with the highest C_max_ values for 4-OH propranolol, p = 0.02. The dotted line represents that Kaplan–Meier survival analysis with the values for the YA dogs (n = 3) removed, p = 0.23. Statistical significance was determined by log-rank (Mantel-Cox) test. **G)** Comparison of propranolol and **H)** 4-OH propranolol C_max_ in YA (age 5, n = 3), OA (age 7 to < 11 years, n = 10), and S (age ^3^ 11 years, n = 6) dogs. Statistical significance was determined by two-tailed unpaired t-test, *p<0.05, **p<0.01, ns=not significant.

To assess propranolol metabolism, we analyzed plasma samples from 19 dogs enrolled in the study, generating drug concentration curves (Fig 3A, B) and calculating the maximum concentrations (C_max_) and total drug exposure (AUC) for propranolol and 4-OH propranolol. The calculated C_max_ and AUC values for each dog are provided in Table S1. On average, the propranolol C_max_ was 18.7 ng/mL with a mean AUC_0–24h_ of 163.7 ng/mL/hour, while 4-OH propranolol had a C_max_ of 13.3 ng/mL and an AUC_0–24h_ of 110.4 ng/mL/hour. To determine whether propranolol’s pharmacokinetics were associated with survival, we performed a correlation analysis between survival and the AUC for propranolol and 4-OH propranolol. No meaningful correlation was observed (p=0.89, r=0.04 and p=0.84, r=−0.05, respectively, and Fig S4A, B). However, we found a correlation between the C_max_ of 4-OH propranolol and long-term survival (Fig 3D, r=−0.50, p=0.028), where long-term survivors had lower C_max_ values compared to short-term survivors. No correlation was observed for propranolol (Fig 3C, r=−0.11, p=0.65). We further validated these findings through a survival analysis, comparing overall survival between dogs with high (top 50%) and low (bottom 50%) C_max_ values for propranolol and 4-OH propranolol (Fig 3E, F). These analyses indicated that long-term survival was associated with lower levels of 4-OH propranolol (Fig 3F, p=0.02); however, this effect appeared to be driven by the young adult dogs, as several older dogs with low 4-OH propranolol C_max_ values had survival times below the median of 134 days (Fig 3D). When the three young adult dogs were excluded from the analysis, this difference was no longer apparent (Fig 3F, p=0.2). Given that propranolol clearance is reduced in older human subjects (55), we next analyzed the C_max_ values for propranolol and 4-OH propranolol across the previously predefined age groups. While the propranolol concentrations were not meaningfully different between the age groups (Fig 3G), the older adult and senior dogs had significantly higher mean C_max_ values for 4-OH propranolol than younger dogs (p=0.003 and p=0.037, respectively; Fig 3H), and the pharmacokinetic profiles were variable in the older dogs (Fig S5). Pharmacokinetic differences between male and female subjects have also been reported in humans, but meaningful differences were not observed the AUC and C_max_ values between the male and female dogs enrolled in the PRO-DOX study (p>0.1). Differences were also not correlated with weight (p>0.2).

These results indicate 4-OH propranolol clearance is higher in younger dogs and varies among older dogs. Although low 4-OH propranolol exposure correlated with long-term survival, faster 4-OH propranolol clearance did not confer a survival advantage as some short-term survivors exhibited comparable metabolism. Overall, propranolol did not appear to improve survival when combined with doxorubicin, as median survival in the PRO-DOX and control groups was similar.

### Higher expression of immune transcripts and lower levels of cell cycle and DNA damage repair transcripts are associated with younger age and improved survival.

**Figure 4:**
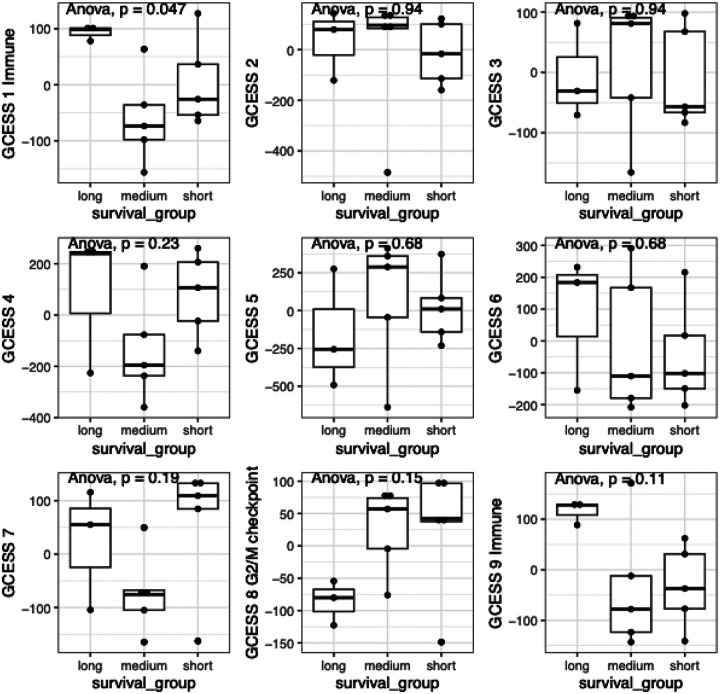
Survival correlates with immune and G2/M checkpoint signatures. Plots show the relationship between immune GCESS (GCESS 1 and 9) and G2/M checkpoint GCESS (GCESS 8) based on survival. Gene expression signatures from the short- (£ 138 days) medium- (^3^ 190 to < 365 days), and long-term (^3^ 365 days) survivors using GCESS. Statistical significance was evaluated using ANOVA.

We next shifted our focus to other age-related biological differences that could contribute to improved survival outcomes observed in the younger dogs. Aging is known to modulate the tumor microenvironment (TME) by reducing immune function, thereby influencing tumor progression and prognosis. To identify differences in gene expression signatures in the tumors of the young adult dogs versus the older adult and senior dogs, we extracted RNA from 13 FFPE tumor samples from dogs enrolled in the PRO-DOX study and analyzed the corresponding RNA-seq data. We used an unbiased method of dimensional reduction, known as GCESS (41), to summarize co-regulated gene clusters in these tumor samples and to derive associations between survival outcomes and transcriptional patterns. This analysis revealed nine distinct clusters, which are depicted in unsupervised hierarchical clustering heatmaps (Fig S6). To assess potential correlations between gene expression and survival, we stratified tumor samples into short-term (≤138 days), medium-term (≥190 to <365 days), and long-term (≥365 days) survival groups. This classification permitted us to equally divide the samples between the medium and short-term survivor groups. The three younger dogs comprised the long-term survivor group, allowing us to also evaluate signatures associated with age. High expression of immune transcripts (GCESS 1 and GCESS 9) and low expression of G2/M checkpoint transcripts (GCESS 8), which include genes involved in cell cycle regulation and DNA damage-induced cell cycle checkpoints, were associated with favorable outcomes in the younger dogs (Fig 4 and Table S2). These results align with a previous study showing that high cell cycle GCESSs and low immune GCESSs from canine and human osteosarcoma and multiple other human cancers are associated with poor survival outcomes (41). For canine osteosarcoma, the G2/M checkpoint was found to be regulated, at least in part, by the retinoblastoma (RB)-E2F1 pathway (58). We noted E2F1 expression within the GCESS 8 cluster, suggesting a role of this regulatory pathway in hemangiosarcoma progression (Table S2).

**Figure 5:**
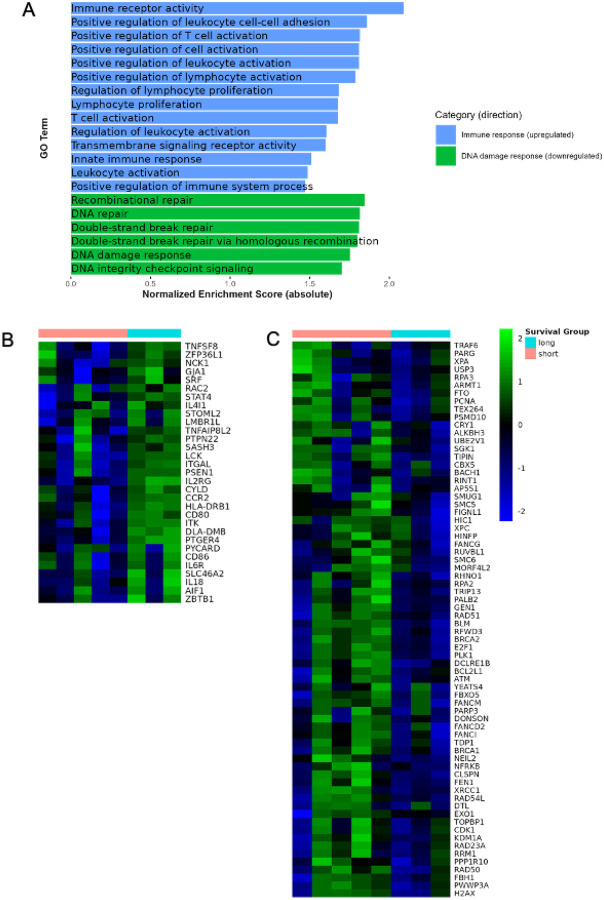
Transcriptional profiling of tumors reveals alterations in genes associated with aging. **A)** Normalized enrichment scores comparing upregulated pathways related to adaptive and innate immunity versus pathways related to DNA damage and the DNA damage response (downregulated) in long-term (“long”) survivors (n=3 YA dogs) and short-term (“short”) survivor groups (n=5, OA and S dogs). Heatmap of differently expressed genes involved in **B)** T cell activation and **C)** DNA damage response.

No individual genes were significantly differentially expressed between young and old dogs, likely due to low statistical power from the small sample size and dog-to-dog variability. To better characterize the biological differences between young adult long-term survivors and short-term survivors (older adult and senior dogs), we performed GSEA on ranked, signed p-values. We tested for Gene Ontology (GO) term enrichment and found numerous enriched GO terms differentiating young adult from older adult dogs (Table S3). In the young adult dogs, upregulated pathways were heavily represented by signatures associated with positive regulation of leukocyte and lymphocyte activation, innate immune responses, and T cell activation (Fig 5A–C). Downregulated pathways were heavily represented by changes in transcripts associated with the cell cycle and the DNA damage response. Collectively, these transcriptional signatures represent conserved prognostic markers across hemangiosarcoma, reflecting both overall survival in response to treatment as well as age-associated tumor progression.

## Discussion

This study highlights the impact of age on hemangiosarcoma outcomes, with younger dogs exhibiting prolonged survival following treatment with doxorubicin-based protocols and suggesting that early-age at diagnosis is a predictor of favorable prognosis. Using gene expression analyses, we identified distinct signatures associated with aging in tumors from young and old dogs. Tumors from young adult dogs exhibited increased expression of markers linked to immune function, while tumors from older dogs showed increased expression of genes associated with the cell cycle and DNA damage and repair. Collectively, these findings highlight several characteristics associated with aging in hemangiosarcoma that can be linked to prognosis and overall survival. Our findings underscore the need for age-specific treatment strategies, particularly for older dogs, where immune-targeted interventions may prolong survival.

Doxorubicin is a widely utilized chemotherapeutic agent in veterinary oncology and is frequently administered as a first-line treatment for hemangiosarcoma (59, 60). While doxorubicin exerts its primary anticancer effects by intercalating between DNA base pairs, disrupting DNA structure, and inhibiting topoisomerase II in dividing cancer cells (61), it also induces immunogenic cell death and stimulates the immune system by promoting the production and activation of CD8 + T cells that specifically recognize and target tumors (62). More recently, doxorubicin has been shown to enhance CD8 + T cell responses in both dogs and humans, consistent with findings from preclinical models (63). In breast cancer patients, increased intratumoral CD8 + T cell levels have been correlated with improved treatment outcomes, reinforcing the role of CD8 + T cell-mediated immunity in the efficacy of anthracycline chemotherapy (64). Similarly, our data demonstrate that tumors from younger dogs exhibit higher expression of genes associated with T cell activation. In contrast, tumors from older dogs appear to have a more immunosuppressive microenvironment, suggesting that age-related changes in immune function contribute to poor treatment responses.

Immunosenescence, including the decline in immune function due to thymic atrophy and increased memory T cells, is a hallmark of aging that impairs immune function and surveillance, reducing the ability of the immune system to detect and eliminate cancer cells (65). During antigen presentation, na’fve CD8 + T cells differentiate into effector T cells, acquiring potent cytotoxic functions that enable direct tumor cell elimination. A subset of these CD8 + T cells differentiate into memory populations, which have a self-replicating capacity and contribute to the maintenance of the T-cell population through differentiation into effector T cells (66). As in older people and mice, the frequency of naïve CD8 + T cells decreases in older dogs along with the proliferative capacity of effector and central memory T cells (67, 68). Increased levels of CD8 + central memory T cells in dogs have also been shown to correlate with aging (69). While memory CD8 + T cells can rapidly develop into cytolytic effector cells and generate antigen specific responses, some memory subsets may also express increased markers of exhaustion, including inhibitory receptors such as PD-1, which contribute to immune dysfunction and exacerbate immunosenescence during aging (70, 71). Blocking the PD-1 pathway has been shown to restore T cell function and expand intratumoral memory CD8 + T cells, in line with research showing that older patients are more responsive to anti-PD-1 therapy (72). While a similar response has not yet been studied in dogs, strategies that reinvigorate antitumor immunity in older dogs could offer a promising avenue for improving outcomes in this group of dogs with hemangiosarcoma.

Increased β-adrenergic receptor (β-AR) signaling has been implicated in the creation of an immunosuppressive TME through receptor expression on immune cells, including macrophages, MDSCs, dendritic cells, and T cells (32, 73). In preclinical models, β-AR activation promoted the expansion of immunosuppressive MDSCs while inhibiting CD8 + T cell activation (74–76). In contrast, antagonism of β-ARs through propranolol or β2-AR deletion reduced the accumulation of MDSCs in spleens and tumor tissue of tumor-bearing mice while increasing T cell infiltration and suppressing tumor growth (74). Propranolol has also been shown to enhance responses to immune checkpoint inhibitors, such as anti-PD-1 and anti-CLTA-4, supporting propranolol as an immune-modulating agent that can reprogram the TME (49, 77). While these findings suggest that propranolol enhances anti-tumor immunity and counteracts immunosuppression, these studies were conducted in young mice, leaving the effects of β-AR blockade on aged immune cells largely unexplored. In adult human subjects, substantially higher levels of b2-AR expression were observed in memory T cell populations compared to naive T cells (78). Furthermore, norepinephrine, a b-AR agonist, was found to suppress activation-induced memory T cell expansion (78). While these findings establish β2-AR expression on memory T cells and a role in immunosuppression, the study cohort spanned individuals from their 20s to 80s without stratification by age, again limiting conclusions regarding age-dependent differences in β-AR expression and the impact of signaling on immune cell activity. Future studies should delineate the role of β-AR signaling across both young and aged immune cell populations from dogs, and whether propranolol or other β-AR antagonists can effectively reverse β-AR-mediated immunosuppression in aging immune systems.

Our findings suggest that long-term survival in younger dogs may be associated with doxorubicin-based therapy, consistent with a similar finding in canine lymphoma (79). However, we cannot fully exclude the potential effects of propranolol on overall survival given the lack of information regarding β-AR expression by canine immune cells. Species-specific differences in propranolol pharmacokinetics also exist between dogs and humans, which further limits our interpretation. Propranolol is a nonselective β-AR antagonist that exists as a racemic mixture composed of the receptor-active S(−) and receptor-inactive R(+) enantiomers (80). In humans, hepatic clearance of the S(−) enantiomer is slower than that of the R(+) enantiomer, with clearance further reduced in individuals over the age of 60 compared to those under 35, leading to higher systemic concentrations of the S(−) enantiomer in older adults (55). In contrast, propranolol metabolism in dogs follows an inverse pattern, where the active S(−) enantiomer exhibits lower serum concentrations than the R(+) enantiomer, resulting in prolonged persistence of the inactive form (57, 81). Both enantiomers are metabolized into several products, including 4-OH propranolol, the primary metabolite in dogs, which is preferentially formed more rapidly from the S(−) enantiomer (57). Although our study did not differentiate between the R(+) and S(−) enantiomers, our data indicate that 4-OH propranolol clearance is increased in younger dogs. Based on prior studies, this altered pharmacokinetic profile likely results in lower systemic concentrations of the active S(−) enantiomer, potentially diminishing CD8^+^ T cell activation or limiting propranolol’s impact on immunosuppressive cell populations such as MDSCs. Optimization of propranolol as an immune-modulatory agent may therefore be warranted. Recently Ammons et al reported that treatment of dogs with brain tumors using propranolol, losartan, and a tumor vaccine achieved a 60% overall biological response rate compared to standard therapy (82). Additionally, pre-treatment of dogs with propranolol prior to autologous dendritic cell administration in dogs with hemangiosarcoma has shown promise and merits further investigation as a potential therapeutic strategy (83).

In addition to reduced expression of genes associated with immune cell function, we also found that tumors from older dogs exhibited increased expression of genes associated with G2/M checkpoint transcripts, which encompassed genes involved in the cell cycle and DNA damage and repair. Damage to DNA triggers the DNA damage response, a highly coordinated network of damage sensors, transducers, and effectors that regulate cell cycle arrest, directing cells toward apoptosis, DNA repair, or a state of permanent cellular cycle arrest known as cellular senescence (84). Cellular senescence, a hallmark of aging, occurs in response to various endogenous and exogenous stresses, including telomere dysfunction, oncogene activation, and persistent DNA damage (85). Previous studies in pre-clinical osteosarcoma models demonstrated that the G2/M checkpoint signature was regulated by the RB-E2F1 pathway (58). E2F1 is a key regulator of cell cycle progression, DNA repair, and senescence (86). It is also highly expressed in cancer cells and linked to poor prognosis in multiple cancers (87–89). This aligns with our findings, where younger dogs with hemangiosarcoma, whose tumors exhibited low expression of G2/M checkpoint transcripts, including E2F1, had significantly longer survival than older dogs with tumors expressing higher levels of these genes. Although this signature was associated with older dogs in our study, osteosarcoma predominantly affects children, adolescents, and young adults (90). Thus, while our findings reinforce the G2/M checkpoint transcriptional signature as a conserved prognostic marker, its role may reflect both poor survival outcomes and age-associated tumor progression across different cancers.

The presence of senescent cells within the TME of older dogs may contribute to immunosuppressive gene signatures and poor treatment responses. These cells continuously secrete senescence-associated secretory phenotype factors, including proinflammatory cytokines, chemokines, growth factors, and proteases, which reshape the TME, promote chronic inflammation, and drive immunosuppression (91). Senescent cells also express immunosuppressive molecules such as PD-L1 and CTLA-4, which inhibit immune cell cytotoxicity. Notably, recent studies indicate that E2F1-driven transcription promotes PD-L1 accumulation in senescent cells, further reinforcing their immunosuppressive role. Therapeutic strategies targeting both the DNA repair machinery and the senescent TME may be particularly effective in older dogs. Combining DNA damage repair inhibitors with immune checkpoint blockade could enhance treatment efficacy by simultaneously disrupting tumor cell survival mechanisms and restoring immune function. Supporting this approach, recent findings in a pancreatic cancer model demonstrated that co-administration of a PARP inhibitor and anti-PD-L1 therapy synergistically reduced tumor growth and significantly increased T cell infiltration (92). Understanding the intricate relationship between aging, DNA damage, and cellular senescence may be essential for developing new, age-specific treatment strategies that improve outcomes in older dogs with hemangiosarcoma.

The observed trend of improved outcomes in younger dogs was largely serendipitous and was limited by the small number of young adult dogs in both the PRO-DOX and the control groups. Our study was also limited by the absence of six-year-old dogs, as none were enrolled in either group, which may have provided additional insight into the transitional effects of aging on treatment outcomes. To validate our findings and support the use of doxorubicin as a standard first-line treatment for young adult dogs with splenic hemangiosarcoma, a dedicated study specifically enrolling dogs under the age of seven is warranted, as well as evaluation of potential age-related differences in doxorubicin pharmacokinetics and pharmacodynamics. Finally, while gene expression analyses revealed that immune signatures associated with the positive regulation of lymphocyte activation were upregulated in tumors from younger dogs, immunohistochemical analysis of CD3 + cells was inconclusive. This may be attributed to tumor heterogeneity and regional variation within tumors, leading to our exclusion of this analysis from the presented data. The gene expression studies, which used five 10-micron curls from each tumor block for RNA extraction, likely captured a broader representation of the tumor microenvironment. Immunohistochemical analyses that incorporate multiple sections across each tumor are likely to be more informative.

## Conclusions

To our knowledge, this is the first study to highlight distinct differences in prognosis and the hemangiosarcoma TME between young and old dogs, revealing several hallmarks of cellular aging within tumors. Additionally, our data suggest a link between DNA damage responses and age-related immune decline in older dogs with shorter survival. These insights provide a strong rationale for further investigating doxorubicin’s effectiveness in younger dogs while developing alternative therapeutic approaches for older dogs that target both DNA repair pathways and immune checkpoint regulation. Tailoring treatment strategies based on age-specific tumor characteristics could improve clinical outcomes and inform future therapeutic development for canine hemangiosarcoma.

## Figures and Tables

**Table 1.** Patient Characteristics

	PRO-DOX	Comparison group	p-value
	Mean ± SD or n (%)	Mean ± SD or n (%)	
**Number of Dogs**	20	40	
**Age at diagnosis (years)**	9.3±2.7	9.8±2.1	0.38
**Sex (male)**	14 (70%)	22 (55%)	0.40
**Stage**			0.65
Stage 1	1 (5%)	5 (12.5%)	
Stage 2	19 (95%)	35 (87.5%)	
**Weight (kilograms)**	33.7±12.3	28.6±11.7	0.13
**Days between splenectomy and chemotherapy**	28.7±6.5	21.8±7.5	0.001
**Median survival**	134	152	0.59

## Data Availability

The datasets used and analyzed for this study are available from the corresponding author on reasonable request.

## Abbreviations

4-OH propranolol: 4-hydroxy propranolol
AE: Adverse event
AUC: Area under the curve
b-AR: beta-adrenergic receptor
CI: Combination index
C_max_: maximum concentration
CPAS: Clinical Pharmacology Analytical Services
CTLA-4: Cytotoxic T-lymphocyte-associated protein 4
DLT: Dose limiting toxicity
FFPE: formalin-fixed paraffin-embedded
GCESS: Gene cluster expression summary score
GO: Gene ontology
GSEA: gene set enrichment analysis
IACUC: Institutional Animal Care and Use Committee
MDSC: Myeloid derived suppressor cell
MN: University of Minnesota
OA: Older adult
PARP: Poly (ADP-ribose) polymerase
PD: Purdue University
PD-1: Programmed death protein 1
PD-L1: Programmed death-ligand 1
PRO-DOX: propranolol combined with doxorubicin
RB: retinoblastoma
REDCap: Research electronic data capture
RIN: RNA integrity numbers
RNA-seq: RNA sequencing
S: Senior
TME: Tumor microenvironment
UMGC: University of Minnesota Genomics Center
UP: University of Pennsylvania
VCOG: Veterinary Cooperative Oncology Group
VMC: Veterinary Medical Center
YA: Young adult
